# Immunohistochemical localization of endothelial nitric oxide synthase in endometrial tissue of women with unexplained infertility

**Published:** 2012-03

**Authors:** Tohid Najafi, Marefat Ghaffari Novin, Jalil Pakravesh, Khadijeh Foghi, Fatemeh Fadayi, Gelareh Rahimi

**Affiliations:** 1 ***Infertility and Reproductive Health Research Center,****Shahid Beheshti University of Medical Sciences, Tehran, Iran.*; 2 *Department of Biology and Anatomical Sciences, Faculty of Medicine, Shahid Beheshti University of Medical Sciences, Tehran, Iran.*; 3 *Infertility Treatment Center, Aban Hospital, Tehran, Iran. *

**Keywords:** *Unexplained infertility*, *eNOS*, *Immunohistochemistry*, *Human endometrium*

## Abstract

**Background:** Nitric oxide (NO) is a molecule that incorporates in many physiological processes of female reproductive system. Recent studies suggested the possible role of endothelial isoform of nitric oxide synthase (eNOS) enzyme in female infertility.

**Objective: **The aim of this study is to evaluate the expression of endothelial nitric oxide synthase in endometrial tissue of women with unexplained infertility.

**Materials and Methods**: In this case-control study a total of 18 endometrial tissues obtained from 10 women with unexplained infertility and 8 normal and fertile women by endometrial biopsy, 6 to 10 days after LH surge. Specimens were fixed in 4% paraformaldhyde fixative and frozen sectioned for semi-quantitative immunohistochemical evaluation using monoclonal anti-human eNOS antibody. Hematoxilin and Eosin was used for Histological dating.

**Results**: Localization of endothelial nitric oxide synthase was seen in glandular and luminal epithelium, vascular endothelium and stroma in both fertile women and women with unexplained infertility. Although there were differences in immunoreactivity of glandular epithelium (p=0.44), vascular endothelium (p=0.60) and stroma (p=0.63) but only over-expression of eNOS in luminal epithelium (p=0.045) of women with unexplained infertility compared to fertile women was statistically significant (p<0.05).

**Conclusion**: This study suggests that changes in luminal expression of eNOS may influence receptivity of endometrium.

## Introduction

Inability to conceive at least for 1 year regularity is determined as infertility (1). In some cases there is not any convincing cause for infertility that comprises up to 30% of infertile couples and named unexplained infertility (2). Traditionally, only one basic infertility evaluations cannot reveal a convincing reason, the diagnosis of unexplained infertility is made. 

However, this evaluation includes updated laboratory tests by the time. Nitric oxide (NO) is a free radical that is derived from L-arginine in the process of its conversion to L-citrullin by the action of nitric oxide synthase (NOS) enzyme (3). This molecule has diverse physiologic functions, including regulation of vascular resistance, participation in cellular injury, and signal transduction (4). 

It has an important role in smooth muscle relaxation so attracted many researches focusing on this field like myometrial NO. Although NOS has been localized in endometrial glands and epithelia, data on its regulation and function in this site particularly in humans are scant and it’s mechanisms of expression in many endometrial-related disorders are unknown. One of earlier reports (5) showed that in the human endometrium, the endothelial isoform of NOS (eNOS) is predominantly expressed and it mainly immunolocalizes to glandular epithelium, although its expression has also been seen in other systems (6). 

Another report revealed that, expression of endometrial eNOS protein and mRNA varies during the menstrual cycle, so that midsecretory phase shows maximal expression (5, 7). Identifying the cause of infertility in unexplained infertility is complex and often faces overlapping etiologies. So, because of the vast incorporation of nitric oxide in biology and physiology of reproduction, we hypothesized that regulation of endothelial nitric oxide synthase may be altered in the process of unexplained infertility, and this can be considered as one of potential causes of inability to conceive. In this study we evaluate localization of endothelial isoform of nitric oxide synthase in endometrial tissues of women with unexplained infertility.

## Materials and methods

**Sample collection and preparation of sections**

Biopsy samples were obtained by using a pipelle curette 6 to 10 days after the urinary LH surge approved by ultrasonography. Control group involved women undergoing total hysterectomy due to their problems other than endocrine, cancer, immune and inflammation -related problems. Women in the control group had normal menstrual cycles (26-33 days) and had not received any hormonal medications for at least 3 months before surgery. Mean age of these women was 35.75 years. All of them (n=8) experienced at least one successful pregnancy without using an assisted reproductive technology. Unexplained infertility cases were unable to conceive for more than one year regularity and all of their laboratory tests such as histerosalpingography, ultrasonography, endocrine and other tests showed normal results (n=10 and mean age =35.1 years). Factors related to infertility like antiphospholipid syndrome and genetical history as well as whom their husbands didn’t show normal sperm analysis results [according to WHO sperm analysis criteria (8)] have been ruled out of study.

At first, obtained samples were fixed in 4% paraformaldhyde tissue fixative for maximum 24h then in 70% ethanol and stored at -20°C until processing. Histological dating was done by using the criteria of Noyes *et al* (9). Each biopsy specimen was later cut into 6µm sections by using a Cryocut and placed on poly l-lysin coated slides and stored in -70°C until immunostaining.

**Immunohistochemistry**

A standard immunohistochemical protocol (horse-radish-peroxidase) was used to visualize the intensity and distribution of eNOS immunostaining. A monoclonal mouse antihuman antibody was used to detect eNOS (ABcam Company, UK). After rinsing with buffer (0.1 M of phosphate-buffered saline with a pH of 7.4), endogenous peroxidases were quenched by incubation in 0.3% H_2_O_2_ in methanol for 15 minutes. Repeated rinses with 0.05% bovine serum albumin in phosphate-buffered saline were performed followed by antigen retrieval using tripsin. The slides were then exposed to 1.5% normal gout serum (DAKO Co., Denmark) in humidified chambers for 30 minutes at room temperature to avoid unspecific bindings of antibody. 

The primary antibody against eNOS (1:100) was placed on slides and incubated at 37°C for 1h. After rinsing for several times with wash buffer, the sections were incubated with the secondary antibody, a rabbit antimouse IgG (ABcam co. UK) diluted 1:1000 in PBS. Incubation with the secondary antibody was performed for 1h at 37°C in incubator. 3, 3-diaminobenzidine in H_2_O_2_ (DAKO co, Denmark) was added to the sections for 15 minutes. Thereafter, the sections were counterstained with hematoxylin and mounted. Negative controls were incubated similarly, but phosphate-buffered saline replaced the primary antibody. We used human full term placental tissue as external positive control as well (9). 

Immunohistochemical staining was evaluated blindly by three independent persons by using a light microscope at -400 magnification. To avoid errors due to uneven staining, two sections of each endometrial biopsy specimen including epithelial, endothelial and stromal cells were evaluated. The staining was graded on a scale of absent, weak, moderate, or strong.

**Statistical analysis**

In this case control study, differences in immunohistochemical staining between control and experiment samples were analyzed by using the Mann Withney tests. Also we used SPSS in case of parametric data of patient’s profiles in their questionnaire. p<0.05 was considered statistically significant.

## Results

**eNOS immunolocalization in endometrium**

**Figure 1:**
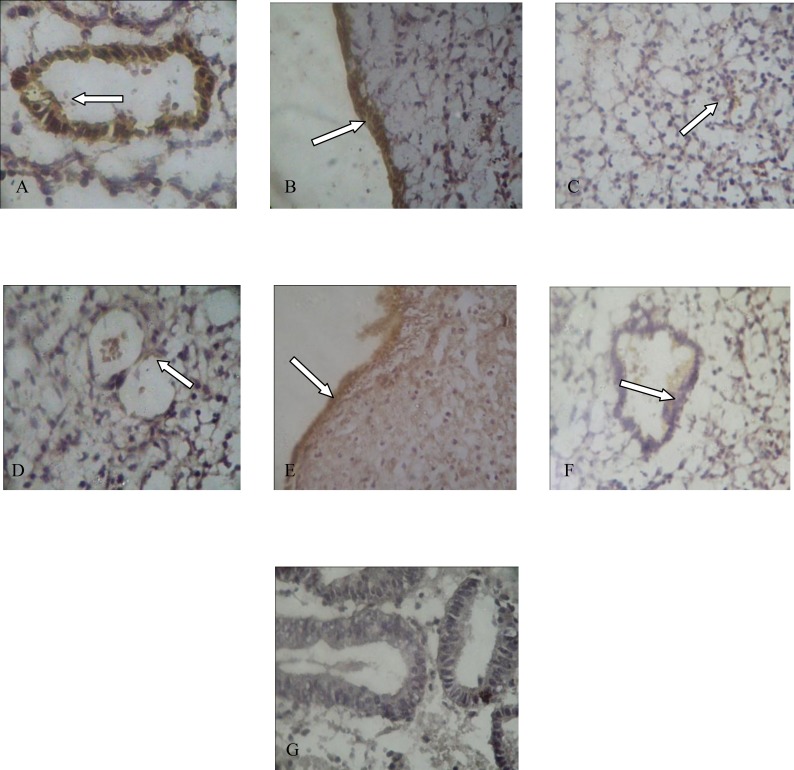
Immunostaining of eNOS in (A) glandular epithelium, (B) luminal epithelium, (C) stroma and (D) vascular endothelium of endometrium with unexplained infertility and luminal (E) and glandular epithelium (F) of normal endometrium. (G) shows staining of glandular epithelium without using primary antibody (negative control). (Original magnification x400).

According to our expectations, endothelial NOS was detected in the vascular endothelium (11), glandular epithelium and luminal epithelium (6), (Figures 1-A, B and D). There was very weak stromal staining for eNOS (Figure 1-C). Immunoreactivity of eNOS was also detected in normal endometrium (Figures 1-E and 1-F). Negative controls didn’t show any immunolocalization of eNOS (Figure1-G). Immunostaining revealed that expression of eNOS in endometrium of infertile women changed in glandular epithelium (p=0.44), luminal epithelium (p=0.045), vascular endothelium (p=0.60) and stroma (p=0.63). 

**Figure 2:**
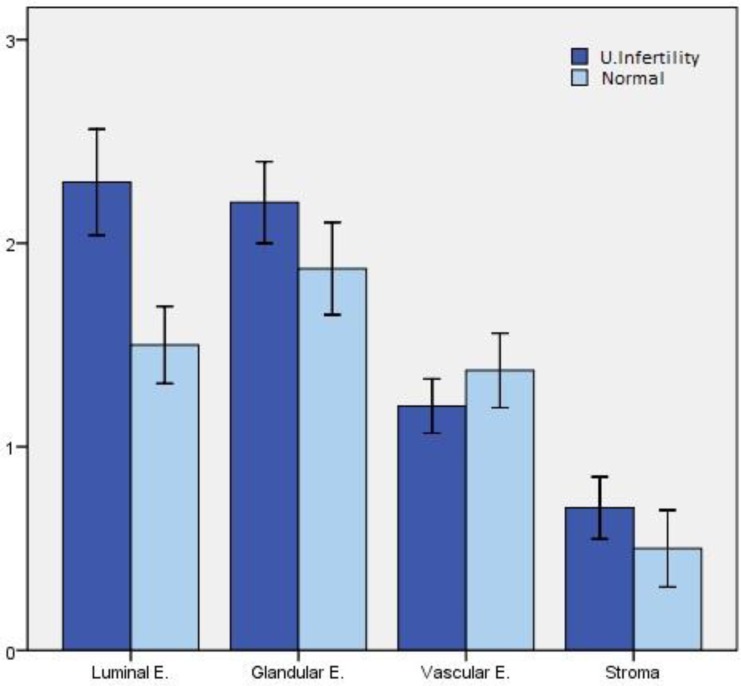
Mean staining of examined four endometrial areas of unexplained infertile women compared with normal endometria. Data were analyzed by Mann Withney U test

According to these data, although there are differences between expression of eNOS in epithelia, stroma and vascular endothelium but these changes cannot be considered significant statistically (p<0.05). However, the difference between two groups in luminal epithelium is statistically considerable (Figure 2).

## Discussion

This study reports that eNOS was localized to blood vessels and was present in glandular and luminal epithelial cells as well as endometrial stroma. These findings are in accordance with previous studies by Tefler *et al* (12). Previous studies by Khorram *et al*, have also shown that eNOS is the main isoform of NOS expressed in the human endometrium at the time of expected implantation (5). 

Glandular epithelium that usually shows higher levels of up-regulation of eNOS, in endometrium of unexplained infertile patients is the most expressed area too. As a result of this expression, like fertile endometrium, the increase in local Nitric Oxide production causes activation of cyclooxygenase-2 and consecutively increase the expression of prostaglandins (13).

Continuous generation of NO into the lumen facilitates processes of menstruation via synthesis of prostaglandin and processes such as implantation via modulation of anchoring proteins. Therefor because of expression of eNOS in luminal epithelium of infertile women, we suggest that prostaglandin and anchoring proteins acting pathways remained intact. eNOS-derived NO can activate soluble guanylyl cyclase generation and enhance cyclo-oxygenase catalysis, thus may act as an alternative inhibitor of endometrial platelet aggregation. 

In this study, we found that eNOS derived NO, is expressed in different areas of endometrial tissues of women with unexplained infertility as of normal endometrium but inconsiderable differences between expressions of eNOS in two groups except in luminal epithelium suggest that endothelial isoform of nitric oxide synthase may not mainly be involved in the process of preparation of endometrium for implantation in unexplained infertility; also endocrine factors acting on stromal cells in this stage would not effected enzyme expression (14). 

The differences in luminal expression of enzyme can hypothesize that process of implantation may be influenced and thus interaction between eNOS and progesterone that regulates various stages of implantation in this area can be changed. This can consequently results in changes in endometrial receptivity. But we can exclude factors acting in L-NAME pathway that inhibit NO synthesis result in prolong duration of delivery in this process. 

Apart from in the vessel wall, the presence of eNOS in glandular epithelium and stromal cells of women with unexplained infertility, suggests that NO may plays its local role in the control of uterine function properly. But we can’t exactly approve that if pregnancy occurs even if for a little period this role could be disrupted. 

It seems that an interrelationship between the cyclo-oxygenase pathway, nitric oxide and cytokines has also been undisrupted (15) as well as proliferation of endometrial epithelial cells (16) and if pregnancy takes place, this complex can regulate uterine function.

In summary, although there are inconsiderable differences in expression of endothelial nitric oxide synthase in stroma, glandular epithelium and vascular endothelium of endometrial tissues of women with unexplained infertility, but changes in expression of enzyme in luminal epithelium suggests that eNOS may be involved in infertility phenomenon of these patients at the stage of implantation, results at least in changes in endometrial receptivity.
